# Significance of Gastric Wall Thickening Detected in Abdominal CT Scan to Predict Gastric Malignancy

**DOI:** 10.1155/2019/8581547

**Published:** 2019-11-20

**Authors:** A. Akbas, H. Bakir, M. F. Dasiran, H. Dagmura, Z. Ozmen, N. Yildiz Celtek, E. Daldal, O. Demir, A. Kefeli, I. Okan

**Affiliations:** ^1^Department of General Surgery, Tokat Gaziosmanpasa University, Tokat 60100, Turkey; ^2^Department of Radiology, Tokat Gaziosmanpasa University, Tokat 60100, Turkey; ^3^Department of Family Medicine, Tokat Gaziosmanpasa University, Tokat 60100, Turkey; ^4^Department of Biostatics, Tokat Gaziosmanpasa University, Tokat 60100, Turkey; ^5^Department of Gastroenterology, Tokat Gaziosmanpasa University, Tokat 60100, Turkey

## Abstract

**Background:**

Early diagnosis of gastric cancer is one of the most important parameters affecting the survival of the disease. In this study, we aimed to stress the importance of antrum wall thickness in CT examination.

**Method:**

The study included 111 patients between ages of 18 and 95 who had antral wall thickening in computed tomography and also had endoscopic evaluation performed in the same clinic. The patients were divided into two groups as benign and malignant according to the pathology results. The thickness of the antrum wall in computed tomography, hemoglobin and albumin levels, and age was compared among these two groups. Parameters with significant differences were further analyzed by multivariate analysis using logistic regression analysis.

**Results:**

Of the 111 patients included in the study, 57 were male and 54 were female. Mean age was 65 years. Fifty-one patients were classified as benign and 60 patients as malignant. Mean age of the malignant patients was 70, while that of benign patients was 59 (*p* < 0.05). Antrum wall thickness was 13.68 ± 3.27 mm in malignant patients and 9.22 ± 2.17 mm in benign patients (*p* < 0.05). Similarly, hemoglobin level was significantly different in malignant and benign patients (10.78 ± 1.57 g/dl and 12.64 ± 1.43 g/dl, respectively; *p* < 0.05). Albumin levels were 3.36 ± 0.57 mg/dl in malignant patients and 3.97 ± 0.57 mg/dl in benign patients (*p* < 0.05).

**Conclusion:**

Evaluation of antrum wall thickness, age, hemoglobin, and albumin values together may contribute to distinguishing the benign and malignant pathologies involving this region in patients with suspected stomach wall thickening in abdominal CT scan.

## 1. Introduction

Gastric cancer is the fourth most common cancer in the world and the third in cancer-related deaths [1, 2]. About 75% of stomach cancer worldwide is observed in Far East countries such as Japan, China, and South Korea [3]. It is twice more common in men than in women and generally occur in 6^th^ and 7^th^ decades [4, 5]. Mortality rates of gastric cancer have declined markedly in recent years [6]. The widespread use of both endoscopic techniques and radiological imaging methods plays an important role in this decline. In countries with high gastric cancer incidence such as Japan and Korea, the rate of early gastric cancer diagnosis has reached up to 50% [7, 8]. In Western society, including Turkey, gastric cancer is diagnosed in more advanced stages. Diagnosis in early stages is one of the most important factors affecting the treatment and survival of the disease [9].

Computed Tomography (CT) is widely used in patients admitted to hospital due to various complaints. CT evaluation by an experienced radiologist may help in the early diagnosis of gastric malignancies. An important issue for the early diagnosis is the ability of CT to differentiate the gastric wall structure and the gastric wall thickness of pathological origins [10]. Increased wall thickness in CT may not always be a sign of malignancy. Gastric wall thickness may also increase due to benign reasons such as gastritis, ulcers, polyps, tuberculosis, Crohn's disease, and Menetrier's disease. Early endoscopy and biopsy evaluations are required to conclude that the wall thickness is due to malignancy [11].

Endoscopy requested due to wall thickness in CT has some major drawbacks such as its high costs, invasive nature, complication risks, and delayed schedules for endoscopic examination. Therefore, timing of endoscopic examination could become critical in patients with wall thickness in CT. There is no common protocol or algorithm to assess these patients presently [11, 12]. The aim of the present study was to determine the association of gastric wall thickening detected in abdominal CT examination requested for various reasons with endoscopic findings and to compare normal and pathological wall thickness.

## 2. Method

This retrospective case-control study was approved by the Ethics Committee of Tokat Gaziosmanpaşa University Faculty of Medicine. Abdominal CT reports of patients who had undergone CT scanning for any reason from 01 January 2012 to 01 August 2018 were searched for the phrase “stomach wall thickening” in the electronic database of the hospital. Patients whose CT scans did not comply with the standard protocol of intravenous and oral contrast matter administration, patients without sufficient distension in stomach, patients with residue food in stomach, patients with congestive heart failure, hypoalbuminemia and nephritic syndrome, patients who had gastric surgery history, patients with anemia history, cases with stomach wall thickness that strongly suggested stomach cancer, and patients who did not have gastroscopy examination in our hospital were excluded. Patients who had oral-IV contrasted abdominal CT in accordance with examination protocols, patients for whom optimum stomach wall thickness could be measured in their CT scans, who had their endoscopic examinations and blood sampling in an interval one month before or after CT examination in our hospital, and who were evaluated using biopsy were included. Abdominal CT sections of the patients included in the study were re-evaluated by an experienced radiologist without being aware of the endoscopic and pathological evaluation results. An increase in gastric wall thickness over 5 millimeters (mm) was considered pathological. Demographic information, hemoglobin (Hb), and albumin values were obtained from electronic files. The cases were divided into two groups as benign or malignant according to the endoscopic and pathological evaluation results. The first group consisted of benign causes such as gastritis, chronic atrophic gastritis, intestinal metaplasia, *Helicobacter pylori* (*H. pylori*) infection, and ulcer, and the second group included malignant causes such as carcinoma, lymphoma, carcinoid, and stromal tumors. Gastric wall thickness, Hb, age, and albumin values of the two groups were compared using descriptive statistics.

Microbiological examination for the presence of *H. pylori* was performed in patients with benign histopathological findings. Differences in *H. pylori* negative and *H. pylori* positive groups were investigated with descriptive statistics. *H. pylori* microbiological studies were not performed for patients with malignant histopathological results.

Descriptive analysis was performed to obtain information about the general characteristics of the study groups. Data for continuous variables were expressed as mean ± standard deviation while data on categorical variables were given as *n* (%). When comparing the quantitative variable means between the groups, independent samples *t*-test and the one-way ANOVA were used. Cross-tables and chi-square tests were used to evaluate the relationships between qualitative variables. *p* values less than 0.05 were considered statistically significant. Logistic regression analysis was performed as multivariate analysis on parameters for which significant differences were observed in univariate analysis. In order to identify significant parameters in multivariate analysis, receiver operating characteristic (ROC) curve analysis was performed. Parameters with cut-off values, sensitivity, and specificity of >0.600 based on area under curve (AUC) calculations were considered significant. SPSS statistical software (ver. 19, SPSS Inc., an IBM Co., Somers, NY) was used in calculations.

## 3. Results

A total of 536 patients were reported to have gastric wall thickness. Four hundred and twenty-five patients who did not comply with the study criteria were excluded from the study. A total of 111 patients (57 males and 54 females) with abdominal CT examination and endoscopic evaluation were included in the study. Histopathological evaluation revealed malignancy in 60 patients (52 adenocarcinomas, 5 lymphomas, 2 gastrointestinal stromal tumors, and 1 neuroendocrine tumor) and benign causes in 51 patients (48 antral gastritis and 3 gastric ulcers).

Gastric wall thickness increase, age, Hb, and albumin levels were significantly different between Groups 1 and 2 in univariate analyses (*p* < 0.05). Based on multivariate logistic regression analysis, suspected gastric wall thickness increase, age, and Hb values were independent variables in the diagnosis of gastric cancer (*p* < 0.05) while albumin was not significant (*p* > 0.05). Characteristics of study groups for these variables are given in Table 1.

**Figure 1:**
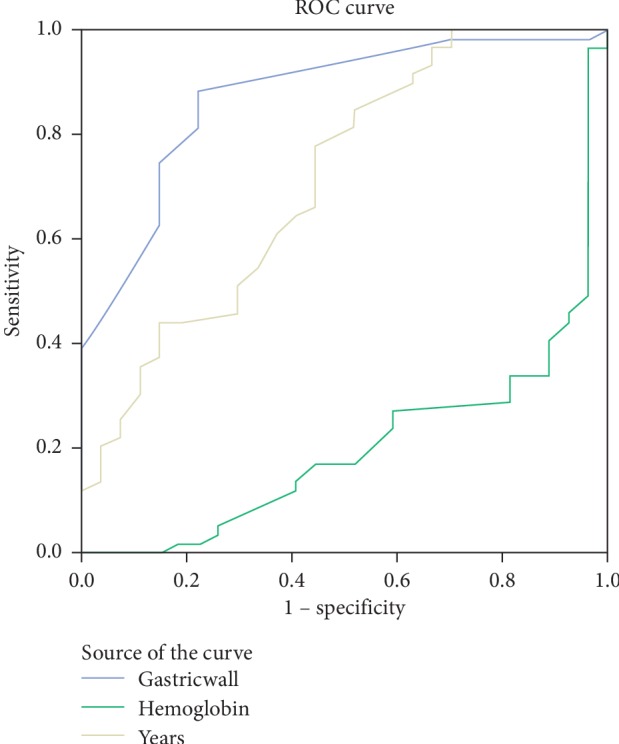
Receiver operating characteristic (ROC) curve analyses of significant parameters for the diagnosis of gastric cancer: gastric wall, hemoglobin, and years (age).

Based on ROC curve analyses of independent variables, AUC values were above 0.600 for antrum wall thickness, hemoglobin, and age (Figure 1). The proposed cut-off values and performance characteristics for these variables are shown in Table 2.

## 4. Discussion

While the five-year survival rate for gastric cancer was 15% in the 1970s, this rate is around 30% nowadays [7]. The most important cause for the poor prognosis is late diagnosis. Importance of abdomen CT scan taken under optimal conditions in the diagnosis of early stomach cancer was emphasized in the final declarations of two important international meetings held in 2014 [13, 14]. The accuracy for the diagnosis of gastric cancer in preoperative CT examinations ranges from 69 to 85%. However, diagnosis is more difficult in early stage gastric cancer cases. Hence, accuracy for the diagnosis is much lower (26–53%) [15, 16].

The five-year survival rate for early stage gastric cancer varies between 85 and 100%. However, this rate is considerably lower for advanced gastric cancer (7–27%) [17]. Increased wall thickness of the stomach in the CT scan is considered pathological in the early diagnosis of gastric cancer. The need for early diagnosis of gastric cancer via CT scan has been the subject of many studies by investigating nonpathological gastric wall thickness. There are many studies reporting that the thickness of normal gastric wall in CT taken under optimal conditions is below 5 mm [18–21]. However, there are also reports indicating that normal gastric wall could be as thick as 12 mm [22].

The most complex location for the evaluation of the gastric wall thickness by CT scan is the antropyloric region (distal portion of the stomach). Increased thickness in the antral wall is mostly attributed to physiological causes (such as excessive peristaltic movements in the antrum and thickness of the smooth muscle wall structure), optimal CT scanning quality coherence (antral distention), or benign causes such as gastritis secondary to *H. pylori* infection. This situation, considered benign and not subjected to further examination, constitutes the major obstacle for early diagnoses of tumors originating from this region. Endoscopic evaluation of all patients with wall thickening in the antropyloric region in CT imaging leads to increased cost, labor loss, complications, and unnecessarily crowded appointment schedule in endoscopy units, which in turn leads to prolonged appointment times and delays in the diagnosis and treatment of veritable patients seeking urgent management [20, 23]. Therefore, a good evaluation of the wall thickness interpreted on CT scan is important in terms of early diagnosis and prevention of unnecessary examinations.

Cho et al. evaluated the antral wall thickness of 120 patients and found an average wall thickness of 12.5 mm arising from benign causes, whereas in cases secondary to malignancy, this was measured to be 19 mm [24]. In another study by Tongdee et al., the antral wall thickness due to malignant causes was 16.64 ± 7.28 mm, and wall thickness due to benign causes was 5.68 ± 2.13 mm [23, 25]. Among the most common causes of benign wall thickness are chronic gastritis and peptic ulcer where *H. pylori* is the major etiologic factor. In developing countries, more than 90% of the population is infected with *H. pylori*, which is 50% in developed countries. *H. pylori* is mostly located in the antrum and is usually asymptomatic [26, 27]. In studies investigating the effect of *H. pylori* on gastric antrum wall thickness, the effect of *H. pylori* positivity was not associated with wall thickness [21, 25]. In the present study, a significant difference was found between the thickness of the antrum wall in benign and malignant groups (OR = 1.60; 95% CI: 1.22–2.09; *p* = 0.01) (Table 1). In ROC analysis, for antrum wall thickness cut-off value of >11 mm, AUC was 0.862, sensitivity was 75%, specificity was 86%, PPV was 0.866, NPV was 0.745, and *p* < 0.001 (Table 2, Figure 1). This could be a good hint to the clinician evaluating abdominal CT scans. Wall thickness could allow early diagnosis and treatment of patients. *H. pylori* positivity was 70.5% in patients who were concluded to be benign based on histopathological examination. In addition, there was no association between mean antrum wall thickness and *H. pylori* positivity (*p* > 0.05).

Gastric cancer is 1.8–2.0 times more common in males than in females. The incidence rate increases with age and is mostly seen in the 6^th^ and 7^th^ decades. Although gastric cancer is usually asymptomatic in early stages, it may cause nausea, vomiting, weight loss, and anemia in advanced stages [28–31]. The symptomatic period of the disease is usually associated with an advanced stage, where malnutrition and chronic anemia are often noticed and confirmed by a decrease in Hb and albumin levels. The mean preoperative Hb values in these patients range from 11.1 g/dl to 12 g/dl [32–35]. Similarly, there are many studies reporting the preoperative albumin values between 3.0 and 3.9 g/dl in patients with gastric tumors [36–38]. In accordance with the literature, there was a significant difference in age, Hb, and albumin values between malignant and benign patient groups in univariate statistical analysis. However, the albumin level was not significant in multivariate logistic regression analysis. Inclusion of patients with suspected wall thickness but exclusion of ones with verified gastric cancer based on CT findings might have resulted in the finding that low albumin level due to malnutrition in our patients was not an independent variable (Table 1). In addition, the benign group included three cases of ulcers. The mean wall thickness of these cases was 13.5 ± 1.29 mm, Hb value was 9.23 ± 1.38 g/dl, and albumin value was 2.97 ± 0.49 mg/dl, which were higher than those in other benign cases and similar to those of the malignant group. Although we classified gastric ulcer as benign, it is among the diseases that should be diagnosed and treated early in terms of the risks involved.

The diagnosis of gastric cancer is based on pathological examination. Therefore, pathological evaluation of endoscopic biopsy is the gold standard in suspected cases. Diagnosis of gastric cancer cannot be made by means of CT imaging, age, Hb, and albumin values alone. Clinicians should be selective when evaluating the patient. Upper endoscopic examination is not recommended for every patient with an epigastric complaint because this can lead to increases in health expenditures, labor loss, complications, unnecessary crowding in endoscopy unit appointments, and delays in the acquisition of health care by patients who need urgent attendance. However, gastric wall thickness, age, albumin, and Hb values could allow clinicians to make a prediction and a preliminary diagnosis when evaluating the patient. We believe that giving priority to the examinations of these specific patients could be effective in decreasing the delays in diagnosis and treatment. In the present study, we observed that gastric wall thickness on CT scan, Hb, age, and albumin values showed significant differences between benign and malignant study groups. These parameters could be useful for the clinician's evaluation of the patient because these parameters may contribute to the diagnosis and treatment of the patient. Larger prospective cohort studies in which gastric wall thickness, Hb, age, and albumin values in addition to clinical symptoms such as loss of appetite, weight loss, nausea, and vomiting should be taken into consideration for the evaluation of patients could be useful to develop a clinical algorithm scheme for the early detection of potentially malignant patients.

Our study carries the drawbacks pertained to all retrospective studies and hence has some limitations. First of all, the study included only patients with suspected gastric wall thickness, but those with normal gastric wall thickness and those who were strongly suspected of gastric tumor in the CT evaluation were excluded. Another limitation was the wide age range. Finally, the limited number of patients may have affected the outcome.

## 5. Conclusion

Antral wall thickness detected in abdominal CT, Hb and albumin levels, and age was significantly different between benign and malignant gastric pathology groups. Hb and age may significantly contribute to patients' outcome by giving priority for upper endoscopic examinations to patients with gastric wall thickness detected on the CT scan. However, further studies are needed to confirm our findings.

## Figures and Tables

**Table 1:** Characteristics of study groups for the parameters investigated.

	Univariate analysis			Multivariate analysis		
	Group 1	Group 2	*p*	OR	95% CI (lower-upper)	*p*
Number of cases	51	60				
Gender			>0.05			
Female	24	30				
Male	27	30				
Antrum wall thickness (mm)	9.22 ± 2.17	13.68 ± 2.28	<0.05	1.60	1.22–2.09	0.01
Hemoglobin (g/dl)	12.64 ± 1.43	10.78 ± 1.57	<0.05	0.58	0.38–0.91	0.02
Age (years)	59.51 ± 15.99	70.37 ± 11.66	<0.05	1.07	1.01–1.13	0.02
Albumin (mg/dl)	3.97 ± 0.57	3.36 ± 0.57	<0.05	0.49	0.09–2.51	0.40

OR = odds ratio.

**Table 2:** The results of ROC (receiver operating characteristic) analysis.

	Cut-off values	Sensitivity	Specificity	PPV	NPV	AUC	*p*
Age (years)	>62	0.783	0.549	0.672	0.683	0.697	<0.001
AWT (mm)	>11	0.750	0.863	0.866	0.745	0.862	<0.001
Hb (g/dl)	≤11.3	0.661	0.889	0.875	0.690	0.796	<0.001

AUC: area under the curve; AWT: antrum wall thickness; Hb: hemoglobin; PPV: positive predictive values; NPV: negative predictive values.

## Data Availability

The data used to support the findings of this study are available from the corresponding author upon request.
